# Interaction of temperature and irradiance effects on photosynthetic acclimation in two accessions of *Arabidopsis thaliana*

**DOI:** 10.1007/s11120-012-9756-3

**Published:** 2012-07-13

**Authors:** Thijs L. Pons

**Affiliations:** Department of Plant Ecophysiology, Institute of Environmental Biology, Utrecht University, Padualaan 8, 3508 CH Utrecht, The Netherlands

**Keywords:** *Arabidopsis thaliana*, Photosynthetic acclimation, Carboxylation capacity, Rubisco, Triose phosphate utilization, Irradiance, Temperature

## Abstract

The effect of temperature and irradiance during growth on photosynthetic traits of two accessions of *Arabidopsis thaliana* was investigated. Plants were grown at 10 and 22 °C, and at 50 and 300 μmol photons m^−2^ s^−1^ in a factorial design. As known from other cold-tolerant herbaceous species, growth of *Arabidopsis* at low temperature resulted in increases in photosynthetic capacity per unit leaf area and chlorophyll. Growth at high irradiance had a similar effect. However, the growth temperature and irradiance showed interacting effects for several capacity-related variables. Temperature effects on the ratio between electron transport capacity and carboxylation capacity were also different in low compared to high irradiance grown *Arabidopsis*. The carboxylation capacity per unit Rubisco, a measure for the in vivo Rubisco activity, was low in low irradiance grown plants but there was no clear growth temperature effect. The limitation of photosynthesis by the utilization of triose-phosphate in high temperature grown plants was less when grown at low compared to high irradiance. Several of these traits contribute to reduced efficiency of the utilization of resources for photosynthesis of *Arabidopsis* at low irradiance. The two accessions from contrasting climates showed remarkably similar capabilities of developmental acclimation to the two environmental factors. Hence, no evidence was found for photosynthetic adaptation of the photosynthetic apparatus to specific climatic conditions.

## Introduction

Photosynthetic acclimation to different levels of growth irradiance has been studied extensively (Boardman 1977; Anderson et al. 1995; Walters 2005). The same is true for growth temperature (Berry and Björkman 1980; Hikosaka et al. 2006; Sage and Kubien 2007). Acclimation to irradiance and temperature is achieved by similar changes in the photosynthetic apparatus, associated metabolism and possibly shared sensory systems (Huner et al. 1998). The two environmental factors could thus interact in their ultimate effect on the photosynthetic apparatus. However, the combined effect of growth irradiance and temperature on photosynthesis has received much less attention in higher plants (Hikosaka 2005; Muller et al. 2005).

Reduced growth irradiance typically causes a reduction in the amount of Rubisco, other Calvin cycle enzymes and components of the electron transport chain, all expressed per unit leaf area. However, chlorophyll content remains generally rather constant (Hikosaka and Terashima 1996), causing a change in the balance between light harvesting and photosynthetic capacity in favor of the former. The change in the balance is achieved by an increase in light harvesting complex (LHC) relative to core chlorophyll, which is reflected in a lower chlorophyll *a*/*b* ratio (Anderson et al. 1995; Hikosaka and Terashima 1995). The reduced photosynthetic capacity relative to light harvesting maintains photon absorption high in the light limited shade conditions, whereas investment in a high photosynthetic capacity would not result in sufficient return as photosynthetic rates are predominantly low. The reduced amount of photosynthetic proteins per area in shade requires a lower number of chloroplasts. This in turn requires less space in mesophyll cells (Terashima et al. 2011), which makes the shade-grown leaf thinner. Shade leaves thus have reduced costs per area in terms of nitrogen (Pons and Anten 2004) and of carbon as the leaf dry mass per area (LMA) is lower (Poorter et al. 2009).

A similar shift in the balance between light harvesting and photosynthetic capacity is observed with variation in growth temperature (Hikosaka et al. 2006). The amount of Rubisco and other components that determine photosynthetic capacity expressed per unit area and per chlorophyll increases at low temperature. This compensates for the reduced activity of the photosynthetic proteins, whereas light harvesting is largely unaffected by temperature (Hikosaka 1997). Acclimation to high growth irradiance and low growth temperature is thus generally reflected in high Rubisco content per unit leaf area and per chlorophyll, a high chlorophyll *a*/*b* ratio and thick leaves (Hikosaka 2005; Muller et al. 2005).

An additional phenomenon associated with acclimation to low growth temperature is increased investment in the capacity of assimilate processing. Warm-grown plants measured at low temperatures typically show inhibition of photosynthesis at high [CO_2_] and/or low [O_2_] (Sage and Sharkey 1987; Atkin et al. 2006; Sage and Kubien 2007). The high rate of production of triose-phosphate by the chloroplast cannot be met by the reduced capacity of its utilization in sucrose synthesis as a result of a lower protein activity at low temperature. This leads to sequestering of phosphate in the cytosol, which limits ATP production in the chloroplast. The limitation of photosynthesis by triose-phosphate utilization (TPU) is avoided in the cold by increasing the capacity of sucrose synthesis (Stitt and Hurry 2002).

The light saturated photosynthetic rate in the absence of limitation by TPU can be limited by two processes. Limitation by the carboxylation capacity of Rubisco at ribulose-bisphosphate (RuBP) saturation (*V*_Cmax_) occurs at low [CO_2_], whereas at higher [CO_2_] the regeneration of RuBP as determined by the electron transport capacity (*J*_max_) limits photosynthesis. The limitation by these two processes can be distinguished in CO_2_ response curves (Farquhar et al. 1980). The *J*_max_ */V*_Cmax_ ratio varies little between species (Wullschleger 1993; Leuning 1997) causing the [CO_2_] where co-limitation by the two processes occurs to be close to the intercellular CO_2_ partial pressure (*C*_i_) at ambient values or somewhat above (Stitt 1991). However, this co-limitation *C*_i_ at light saturation is temperature dependent, since *J*_max_ increases stronger with temperature than the initial slope of the CO_2_ response curve, which is determined by *V*_Cmax_ (Kirschbaum and Farquhar 1984). The relative constancy of the initial slope with temperature is caused by the increasing Michaelis–Menten constant of Rubisco and the increasing oxygenation to carboxylation ratio with increasing temperature. Several plants adjust the *J*_max_ */V*_Cmax_ ratio by increasing it (measured at a common temperature) with decreasing growth temperature (Hikosaka et al. 1999), causing a homeostatic tendency in the co-limitation *C*_i_, but not all species do so (Onoda et al. 2005). The adjustment contributes to efficient utilization of resources that are devoted to *J*_max_ and *V*_Cmax_.

The photosynthetic growth irradiance responses as described above has also been documented for *Arabidopsis thaliana* (Walters 2005) and cold and warm temperature effects on photosynthetic performance have been extensively investigated as well (Stitt and Hurry 2002). These studies showed that *Arabidopsis* is very well capable of acclimation to shade and cold. The latter is not surprising since most of its populations exhibit a winter annual life history (Mitchell-Olds and Schmitt 2006), which means that much of its growth occurs in the cool season. However, the possible interacting effects of growth temperature and irradiance on photosynthetic characteristics have not been investigated in this or in other species.

The first question to be addressed is to what extent the effect on photosynthetic acclimation of growth temperature depends on growth irradiance and vice versa. It is hypothesized that the two factors may interact, since several aspects of photosynthetic acclimation are shared. To investigate the interaction, *Arabidopsis* was grown at two levels of irradiance and temperature in a factorial design. Since the plants were grown in constant conditions, developmental acclimation is addressed here as distinguished from dynamic acclimation in response to a change in growth conditions that is regulated differently (Athanasiou et al. 2010).

*Arabidopsis thaliana* has a large geographical distribution (Koornneef et al. 2004) involving substantial climatic variation. Intraspecific variation in capability of photosynthetic acclimation to irradiance and temperature is known from other species (Björkman and Holmgren 1963; Pearcy 1977; Flood et al. 2011). This has not been investigated in *Arabidopsis*. The second question to be addressed is whether intraspecific variation in the capability of photosynthetic acclimation to temperature and irradiance exists in *Arabidopsis*. It is hypothesized that such variation is present in two accessions from contrasting latitudes. Accessions from the Cape Verde Islands and from Finland were included in the study as a first investigation of possible climatic adaptation of the photosynthetic apparatus to the local climate in *A.* *thaliana*.

## Materials and methods

### Plant material and growth conditions

Two accession of *A.* *thaliana* L. were used for the experiment (Nothingham *Arabidopsis* Stock Centre), CVI-0 (N902) collected on the Cape Verde Islands (15°N; −24°E) and Hel-1 (N1222) collected in Finland near Helsinki (60°N; 25°E). Climate data for the collection sites were obtained from the Royal Dutch Meteorological Institute (KNMI) climate explorer (http://climexp.knmi.nl; ERA reanalysis). Mean annual temperature is a rather constant 24 °C throughout the year for Cape Verde Islands at sea level. CVI-0 was collected at 1200 m altitude, causing the mean temperature to be about 15 °C with day temperature several degrees higher. Leaf temperatures are likely to be high in sunny conditions for this small rosette growing close to the soil surface. In Helsinki, mean annual temperature is 10 °C for the months with mean temperatures above zero (April–November) with large seasonal variation, low in autumn and spring during vegetative growth and higher towards summer with the transition to flowering and seed set. Mean photosynthetically active irradiance (400–700 nm) is 1,120 and 510 μmol photons m^−2^ s^−1^, assuming 12- and 14-h day length for Cape Verde and Helsinki for the above zero temperature months, respectively. Irradiance at the level of the small plants is likely to be lower than the values given above as a result of shading by surrounding plants and objects.

The plants were grown hydroponically in a growth chamber at 70 % relative humidity. Light was provided during an 8 h photoperiod with fluorescent (Osram-L 20SA 140 watt) and incandescent lamps (Philips 60 watt). Seeds were incubated for 4 days at 4 °C in a Petri dish and thereafter germinated at 20 °C. The germinated seeds were planted in the growth chamber in Eppendorf tubes with lid and bottom removed and filled with expanded clay granules topped with rockwool. When the roots started to grow through the open bottom, the tubes were transferred to a container with a diluted nutrient solution containing 2 mM NO_3_^−^ with other nutrient elements in proportion (Poorter and Remkes 1990), kept at pH 5.8 and renewed weekly. The chamber was divided in two compartments with different photosynthetic irradiance, 300 and 50 μmol photons m^−2^ s^−1^. The temperature was first set at 22 °C for growing plants at high temperature and subsequently at 10 °C for growing plants at low temperature.

We aimed to measure the fully grown sixth leaf. However, the plants were growing very slowly in the cold at low irradiance. Hence, the fifth leaf was used in these plants. The plants were measured at ~4 weeks after germination at high temperature and high irradiance (HTHL), 6 weeks at high temperature and low irradiance (HTLL), 7 weeks at low temperature and high irradiance (LTHL) and 9 weeks at low temperature and low irradiance (LTLL).

### Photosynthesis measurements

The CO_2_ response of photosynthesis was measured with small leaf chambers, custom made for containing whole *Arabidopsis* leaves (window 27 × 60 mm). A fan kept the boundary layer conductance high (around 7 mol m^−2^ s^−1^ depending on leaf size). Three chambers were used simultaneously (*n* = 3 for the CO_2_ response) in a system as described previously (Pons and Welschen 2002). They were connected to a temperature regulated water bath and could be alternately connected to an IRGA (Licor 6262, Lincoln, Nebraska, USA) for measuring the gas exchange rates. Light was provided by means of slide projectors with a halogen lamp. The leaves were kept in the leaf chamber at saturating irradiance as derived from irradiance response curves (1,000 and 300 μmol photons m^−2^ s^−1^ for HL- and LL-plants, respectively) and ambient [CO_2_] until steady state gas exchange rates were achieved (at least 30 min). Thereafter the CO_2_ response was measured from low to high [CO_2_] with three CO_2_ concentrations below ambient and three above. Measurements were done with the leaf temperature set at the two growth temperatures (10 and 22 °C). The CO_2_ compensation point in the absence of respiration in the light (Γ*) was estimated at the two temperatures on *Arabidopsis* Col-0 plants grown at 20 °C using the Brooks and Farquhar (1985) method. Atmospheric pressure was 101.6 kPa on average.

The temperature dependence of net CO_2_ assimilation rates at ambient [CO_2_] (38 Pa) and at the growth and saturating irradiance (*A*_growth_ and *A*_sat_, respectively) was measured in two Parkinson leaf chambers. The chambers were modified so that they could be connected to the same system as mentioned above (Pons and Welschen 2002). The measurements were done twice with the two chambers (*n* = 4). The chamber with a circular window of 2.5 cm^2^ was used to simultaneously measure gas exchange and chlorophyll fluorescence (PAM-2000; Walz, Germany). Measurements were done at ambient [O_2_] (21 %) and low [O_2_] (1 %) in order to estimate the degree of limitation by TPU (Sage and Sharkey 1987). Gas exchange data for both chamber types were corrected for minor leakages using empty chamber values and in the case of the Parkinson chambers also for dark respiration of leaf parts clamped under the gasket (Pons and Welschen 2002).

### Structural and chemical analysis

After the measurements leaf punches of 0.126 cm^2^ were sampled for measuring chlorophyll, two in the case of small leaves (<3 cm^2^) and four when leaves were larger. The remainder of the leaves from the CO_2_ response measurements was used for measuring Rubisco content. The remainder of the leaves from the temperature response measurements was used for determining LMA from leaf dry mass and area.

Rubisco contents were measured as described previously (Westbeek et al. 1999; Mommer et al. 2005). The leaf extract was run on SDS-PAGE gels that were scanned. Custom-made image analysis was used to calculate Rubisco content from the large subunit. Chlorophyll was extracted in dimethylformamide (DMF) for at least 5 days in darkness. Contents were calculated using the formula provided by Inskeep and Bloom (1985).

### Calculations

The CO_2_ response data were used to calculate carboxylation capacity (*V*_Cmax_), electron transport capacity (*J*_max_) and the intercellular CO_2_ partial pressure (*C*_i_) where co-limitation between these capacity variables occurred using the Farquhar et al. (1980) model. Γ* values obtained from our own measurements were, 21.3 and 37.0 mol mol^−1^ for 10 and 22 °C respectively. Values for in vivo Rubisco kinetics parameters *k*_*c*_ and *k*_*o*_, 40.1 Pa and 27.59 kPa at 25 °C, and their temperature dependence were obtained from Bernacchi et al. (2002). Distinction between *V*_Cmax_ limited, *J*_max_ limited and TPU limited *C*_i_ trajectories was done by eye. The model was fitted to the data using the solver module in Excel 2007 for the *V*_Cmax_ and *J*_max_ limited *C*_i_ ranges only.

Electron transport rate (ETR) was calculated according to Genty et al. (1989) from the photochemical efficiency in the light ($\varphi_{\text{II}} = \Updelta F/F_{\text{m}}^{\prime }$) as measured by chlorophyll fluorescence, photon flux density (PFD) and leaf absorptance (abs) as ETR = *φ*_II_ PFD abs 0.5. Absorptance was estimated from the chlorophyll content (chl) as abs = chl/(chl + 76) (Evans and Poorter 2001).

Data are presented as means with standard deviation (SE). The SE was calculated as the standard deviation divided by the square root of the sample size (*n*). Further statistical analysis was by three-way ANOVA using accession, growth temperature and growth irradiance as fixed factors (SPSS 18.0). All variables were log10 transformed prior to analysis in order to investigate relative effects and to obtain a better homogeneity of variances. Only variables that were already relative expressions were not transformed (chlorophyll *a*/*b* ratio, *C*_i_ */C*_a_ ratio, and O_2_ sensitivity of *A*_growth_ and ETR).

## Results and discussion

The two *Arabidopsis* accessions showed remarkably similar responses to growth temperature and irradiance for many of the variables (Table 1). Therefore, the comparison between the accessions is addressed at the end of this section, where also possible implications for climate adaptation are discussed.

**Table 1:** Results of a 3-way ANOVA for variables shown in the Figures and Table 2

	Accession	Temp.	Light	A × T	A × L	T × L	A × T × L
Fig. 1							
*A*_sat_/LA 10 °C	7.4*	320***	934***	1.9^ns^	0.0^ns^	0.8^ns^	0.8^ns^
*A*_sat_/LA 22 °C	0.0^ns^	79.9***	403***	0.5^ns^	0.4^ns^	18.7***	0.9^ns^
*A*_growth_/LA 10 °C	5.8*	213***	1162***	0.2^ns^	0.9^ns^	13.1**	0.4^ns^
*A*_growth_/LA 22 °C	3.2^ns^	10.1**	1855***	0.3^ns^	0.0^ns^	2.4^ns^	0.1^ns^
ETR/LA Lgrowth 10 °C	4.5*	138***	5062***	9.0**	0.9^ns^	26.1***	0.7^ns^
ETR/LA Lgrowth 22 °C	3.0^ns^	21.4***	17965***	8.5**	3.9^ns^	2.9^ns^	0.1^ns^
ETR/LA Lsat 10 °C	2.0^ns^	140***	660***	6.1*	1.2^ns^	0.4^ns^	0.3^ns^
ETR/LA Lsat 22 °C	0.6^ns^	90***	977***	7.3*	0.7^ns^	8.8**	0.1^ns^
Fig. 3							
*V*_Cmax_/Rubisco 10 °C	0.5^ns^	6.1*	26.7***	0.9^ns^	5.9*	0.1^ns^	0.0^ns^
*V*_Cmax_/Rubisco 22 °C	0.5^ns^	1.0^ns^	43.5***	2.5^ns^	11.0**	6.4*	0.1^ns^
Fig. 4							
*C*_i_ at co-limitation 22 °C	0.6^ns^	5.9^ns^	3.0^ns^	0.6^ns^	1.2^ns^	50.7***	0.2
Fig. 5, O_2_ sensitivity							
ETR 10 °C	1.2^ns^	202***	71.2***	1.6^ns^	0.5^ns^	79.9***	0.0^ns^
ETR 22 °C	0.0^ns^	0.7^ns^	9.2**	4.5*	0.1^ns^	0.2^ns^	1.3^ns^
*A*_growth_ 10 °C	3.0^ns^	178***	13.3**	0.5^ns^	1.8^ns^	10.0**	1.7^ns^
*A*_growth_ 22 °C	0.7^ns^	14.4***	0.2^ns^	3.6^ns^	8.6**	15.3***	9.8**
Table 2							
LMA	11.8**	152***	1121***	23.4***	3.7^ns^	5.2*	0.5^ns^
Chlorophyll/LA	5.1*	43.6***	93.6***	47.2***	0.2^ns^	1.6^ns^	0.0^ns^
Chlorophyll *a*/*b*	10.0**	134***	379***	4.8*	3.9^ns^	17.0***	12.2**
Rubisco/LA	0.0^ns^	18.2***	60.7***	0.5^ns^	0.2^ns^	0.8^ns^	0.9^ns^
Rubisco/chl	0.7^ns^	11.4**	43.4***	1.3^ns^	0.0^ns^	2.4^ns^	1.4^ns^
*A*_sat_/chl 10 °C	23.7***	327***	994***	21.3***	0.0^ns^	4.1^ns^	3.9^ns^
*A*_sat_/chl 22 °C	0.2^ns^	52.0***	310***	4.6*	0.4^ns^	26.1***	0.4^ns^
*V*_Cmax_/LA 10 °C	1.5^ns^	129***	469***	7.0*	6.6*	3.7^ns^	2.7^ns^
*V*_Cmax_/LA 22 °C	1.4^ns^	94.2***	584***	12.6**	12.8**	26.4***	5.3*
*V*_Cmax_/chl 10 °C	6.3*	89.4***	360***	0.1^ns^	15.4**	8.2*	3.1^ns^
*V*_Cmax_/chl 22 °C	7.8*	65.2***	556***	0.3^ns^	31.6***	52.0***	7.6*
*J*_max_/*V*_Cmax_ 22 °C	0.4^ns^	5.3^ns^	2.4^ns^	0.4^ns^	0.9^ns^	48.8***	0.1^ns^
*C*_i_/*C*_a_ Lgrowth 10 °C	1.1^ns^	0.6^ns^	12.5**	13.0**	0.3^ns^	0.3^ns^	0.2^ns^
*C*_i_/*C*_a_ Lgrowth 22 °C	0.0^ns^	5.8*	23.2***	5.6*	1.8^ns^	10.4**	1.5^ns^
*g*_s_ Lgrowth 10 °C	0.6^ns^	19.7***	87.4***	5.6*	0.7^ns^	0.6^ns^	2.0^ns^
*g*_s_ Lgrowth 22 °C	0.2^ns^	2.3^ns^	145***	1.5^ns^	3.5^ns^	5.9*	0.0^ns^

**Fig. 1:**
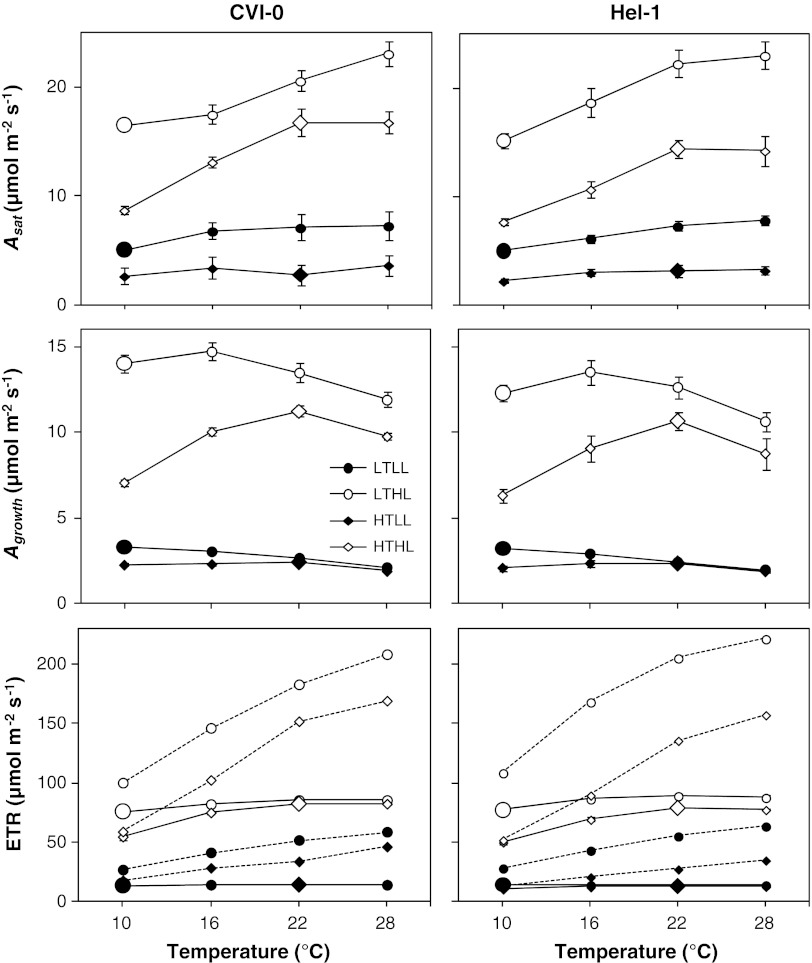
Temperature effects on photosynthesis per unit leaf area of leaves of two *Arabidopsis* accessions (CVI-0 and Hel-1) grown at temperatures of 10 and 22 °C and irradiances of 50 and 300 μmol photons m^−2^ s^−1^. The light saturated rate of CO_2_ assimilation (*A*_sat_), the net CO_2_ assimilation rate at the growth irradiance (*A*_growth_), and the electron transport rate (ETR) at the growth irradiance (continuous line) and at saturating irradiance (dashed line) are shown. Means (*n* = 4) are shown, in the case of *A*_sat_ and *A*_growth_ with SE but for ETR without. Abbreviations of the treatments as indicated in the legend are LTLL (low temperature and low irradiance), LTHL (low temperature and high irradiance), HTLL (high temperature and low irradiance), HTHL (high temperature and high irradiance). Large symbols refer to measurements at the growth temperature

For the effects of measurement temperatures in Figs. 1 and 5, only 10 and 22 °C are depicted. *F* values are shown and probability levels (degrees of freedom = 1) are indicated as ^ns^ *P* > 0.05, * *P* < 0.05, ** *P* < 0.01, *** *P* < 0.001

*A*_growth_ rate of photosynthesis at the growth irradiance, *A*_sat_ light saturated rate of photosynthesis, ETR electron transport rate, LMA leaf mass per area, *V*_Cmax_ carboxylation capacity, *J*_max_ electron transport capacity, *C*_i_ intercellular CO_2_ partial pressure, *g*_*s*_ stomatal conductance for water vapor, *Lgrowth* at the growth irradiance, *Lsat* at saturating irradiance, *LA* leaf area, *chl* chlorophyll

### Photosynthesis per unit leaf area

Increasing growth irradiance caused an increase in the light saturated rate of photosynthesis (*A*_sat_) (Fig. 1; Table 1). This is well known for *Arabidopsis* (Walters and Horton 1994; Walters et al. 1999; Bailey et al. 2004; Boonman et al. 2009) and most other species (Boardman 1977; Walters 2005). Decreasing growth temperature also increased *A*_sat_ when measured at a common temperature (Fig. 1; Table 1). This is also well known from other studies with *Arabidopsis* (Strand et al. 1997; Stitt and Hurry 2002; Bunce 2008; Gorsuch et al. 2010) and with many other species (Berry and Björkman 1980). It resulted in an even larger *A*_sat_ at the growth temperatures in LT-plants compared to HT-plants measured at the growth temperature (Fig. 1). This tendency for homeostasis or even overcompensation is typical for cold-tolerant fast-growing species (Atkin et al. 2006; Yamori et al. 2009). Growth temperature and irradiance were not acting fully independently, as relative effects on *A*_sat_ were stronger in LL-plants compared to HL-plants when measured at 22 °C but not at 10 °C (Fig. 1; Table 1).

Temperature optima for photosynthesis at the growth irradiance (*A*_growth_) were lower compared to the optima for *A*_sat_ (Fig. 1). *A*_growth_ was light limited and thus also limited by electron transport for most of the temperature range, except the lowest temperature, as evident from the ETR measurements (Fig. 1). This makes the ETR at the growth irradiance independent of temperature. However, increasing temperature increases the proportion of oxygenation reactions of Rubisco and thus decreases net photosynthesis over the light limited range (Berry and Björkman 1980; von Caemmerer 2000) (Fig. 1). The effect is stronger for LT-plants due to their higher *A*_sat_, particularly at low temperatures, causing a lower optimum temperature for *A*_growth_ in these plants. The light limitation was stronger at low compared to high growth irradiance, causing an even lower temperature optimum in LL-plants and a smaller relative growth temperature effect on *A*_growth_ and ETR measured at 10 °C compared to HL-plants (Fig. 1; Table 1).

The stomatal conductance (*g*_s_) under growth conditions was high relative to *A*_growth_, resulting in a rather high ratio of intercellular to atmospheric [CO_2_] (*C*_i_/*C*_a_) of 0.84 (Table 2). This is generally found in hydroponically grown plants (Poorter and Evans 1998). The *g*_s_ was lower in LL- compared to HL-plants, whereas *C*_i_/*C*_a_ was slightly higher as is often the case (Poorter and Evans 1998). The growth temperature effect on *C*_i_/*C*_a_ was less consistent and showed small differences between the two accessions and some interaction with irradiance (Tables 1, 2). The small variation in *C*_i_/*C*_a_ was of little importance for the variation in *A*_growth_.

**Table 2:** Structural, chemical, and gas exchange variables (mean ± SE) of *Arabidopsis* leaves from two accession (CVI-0 and Hel-1) grown at temperatures of 10 and 22 °C and irradiances of 50 and 300 μmol photons m^−2^ s^−1^

Accession	CVI-0				Hel-1			
Growth temperature	10 °C		22 °C		10 °C		22 °C	
Growth irradiance (μmol m^−2^ s^−1^)	50	300	50	300	50	300	50	300
LMA (g m^−2^)	10.8 ± 0.3	32.2 ± 1.0	9.1 ± 0.5	24.6 ± 0.7	11.7 ± 0.5	32.3 ± 1.0	7.7 ± 0.5	17.9 ± 0.4
Chlorophyll/LA (μmol m^−2^)	218 ± 9	294 ± 7	226 ± 7	288 ± 11	280 ± 7	371 ± 13	203 ± 7	252 ± 16
Chlorophyll *a*/*b* (mol mol^−1^)	3.40 ± 0.03	4.98 ± 0.08	3.07 ± 0.05	3.82 ± 0.10	3.41 ± 0.01	4.39 ± 0.07	2.93 ± 0.02	3.85 ± 0.04
Rubisco/LA (μmol m^−2^)	1.50 ± 0.14	3.80 ± 0.08	1.04 ± 0.18	2.56 ± 0.30	1.93 ± 0.31	3.47±0.14	0.92 ± 0.20	2.49 ± 0.41
Rubisco/chl (mmol mol^−1^)	7.20 ± 0.51	12.32 ± 0.59	4.79 ± 0.67	8.71 ± 0.99	6.85 ± 0.95	9.37 ± 0.31	4.50 ± 0.78	9.79 ± 0.58
*A*_sat_/chl (mmol mol^−1^ s^−1^)								
10 °C	**22.4** **±** **0.3**	**56.6** **±** **1.7**	11.5 ± 0.7	28.0 ± 0.4	**17.9** **±** **0.3**	**40.6** **±** **1.9**	10.7 ± 0.5	30.7 ± 2.4
22 °C	31.3 ± 1.2	70.6 ± 3.4	**11.9** **±** **0.9**	**55.6** **±** **1.3**	26.7 ± 1.1	59.6 ± 3.7	**15.0** **±** **2.3**	**57.5** **±** **5.3**
*V*_Cmax_/LA (μmol m^−2^ s^−1^)								
10 °C	**9.8** **±** **0.6**	**31.1** **±** **4.0**	5.6 ± 0.5	18.5 ± 1.5	**10.0** ± **0.1**	**35.7** ± **1.1**	3.5 ± 0.5	18.8 ± 1.1
22 °C	26.8 ± 1.3	74.4 ± 2.5	**16.0** **±** **0.9**	**61.5** **±** **2.9**	28.5 ± 0.2	91.8 ± 4.5	**8.9** **±** **1.4**	**66.0** **±** **5.8**
*V*_Cmax_/chl (mmol mol^−1^ s^−1^)								
10 °C	**47.1** **±** **1.7**	**99.9** **±** **5.9**	26.4 ± 2.8	62.9 ± 4.8	**35.9** **±** **1.0**	**96.7** **±** **6.5**	17.3 ± 1.7	75.8 ± 5.2
22 °C	129.6 ± 8.7	240.7 ± 8.8	**74.3** **±** **2.7**	**209.0** **±** **7.5**	102.0 ± 2.9	249.4 ± 21.7	**43.7** **±** **4.6**	**263.8** **±** **9.6**
*J*_max_ */V*_Cmax_ (mol mol^−1^)								
10 °C	**3.23** **±** **0.02**	**3.17** **±** **0.08**	High^a^	Low^b^	**3.27** **±** **0.06**	**3.08** **±** **0.05**	High^a^	Low^b^
22 °C	2.08 ± 0.10	2.51 ± 0.08	**2.26** **±** **0.02**	**2.06** **±** **0.09**	2.08 ± 0.02	2.39 ± 0.04	**2.24** **±** **0.03**	**2.04** **±** **0.03**
*g*_s_ at growth L (mmol m^−2^ s^−1^)								
10 °C	**140** **±** **20**	**304** **±** **22**	65 ± 7	162 ± 10	**80** **±** **8**	**293** **±** **57**	83 ± 14	181 ± 23
22 °C	111±13	249 ± 19	**89** **±** **8**	**343** **±** **61**	85 ± 10	275 ± 12	**93** **±** **20**	**475** **±** **47**
*C*_i_/*C*_a_ at growth L								
10 °C	**0.90** **±** **0.00**	**0.82** **±** **0.01**	0.84 ± 0.01	0.79 ± 0.02	**0.81** **±** **0.02**	**0.76** **±** **0.04**	0.88 ± 0.02	0.83 ± 0.01
22 °C	0.89 ± 0.01	0.79 ± 0.01	**0.86** **±** **0.01**	**81** **±** **0.02**	085 ± 0.02	0.76 ± 0.01	**0.86** **±** **0.03**	**0.87** **±** **0.00**

Gas exchange variables were measured at 10 and 22 °C. Data shown in bold refer to measurements at the growth temperature and irradiance. For abbreviations and symbols see Table 1

^a^Limitation of CO_2_ assimilation by *J*_max_ was not evident in all replications. *J*_max_/*V*_Cmax_ and *C*_i_ at co-limitation are thus high, but could not be reliably estimated

^b^Limitation of CO_2_ assimilation by TPU occurred at low *C*_i_. This prohibited the estimation of *J*_max_. The *J*_max_ */V*_Cmax_ ratio was thus low, but could not be quantified

**Fig. 2:**
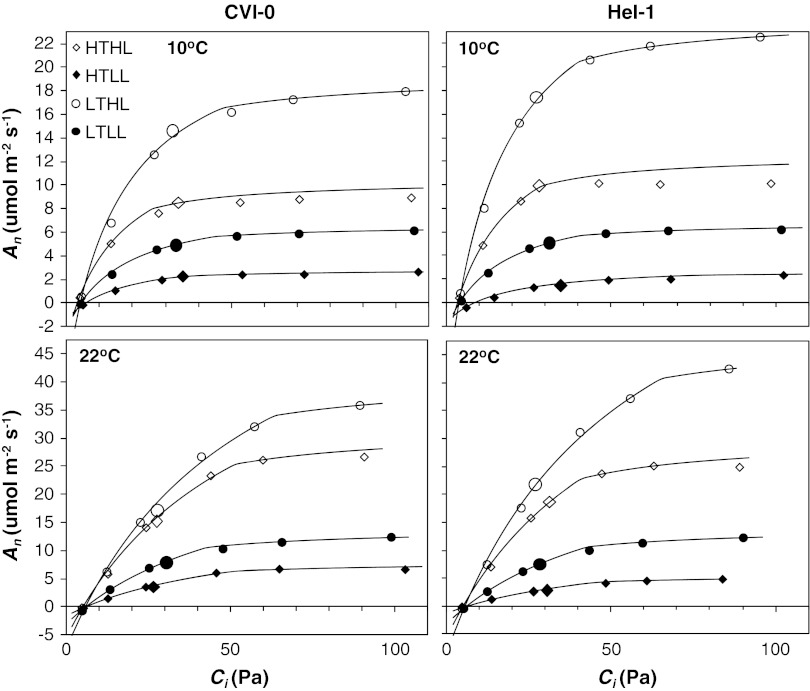
The response of net photosynthesis (*A*_n_) to the intercellular CO_2_ partial pressure (*C*_i_) measured at 10 °C (*upper panels*) and 22 °C (*lower panels*). A representative example (from *n* = 3) is shown for all treatment combinations and the two *Arabidopsis* accessions CVI-0 and Hel-1. *Large symbols* refer to measurements at ambient CO_2_ (38 Pa). The data were fitted to the model of Farquhar et al. (1980) to derive values for *J*_max_ and *V*_Cmax_ and to draw the *lines* as shown

The CO_2_ response of net photosynthesis at light saturation shows that the transition from the *C*_i_ range limited by Rubisco activity at RuBP-saturation to the RuBP-limited range, the *C*_i_ where these processes are co-limiting, was above *C*_i_ at ambient CO_2_ under the growth conditions (Fig. 2). *A*_sat_ is thus Rubisco-limited at light saturation and at the growth temperature as is generally the case (Stitt 1991). Not surprisingly, carboxylation capacity (*V*_Cmax_) as derived from the CO_2_ response showed a similar growth temperature and irradiance dependence as *A*_sat_ (Tables 1, 2). However, the Rubisco content per unit leaf area showed a smaller effect of growth irradiance and no interaction with growth temperature was found (Tables 1, 2).

**Fig. 3:**
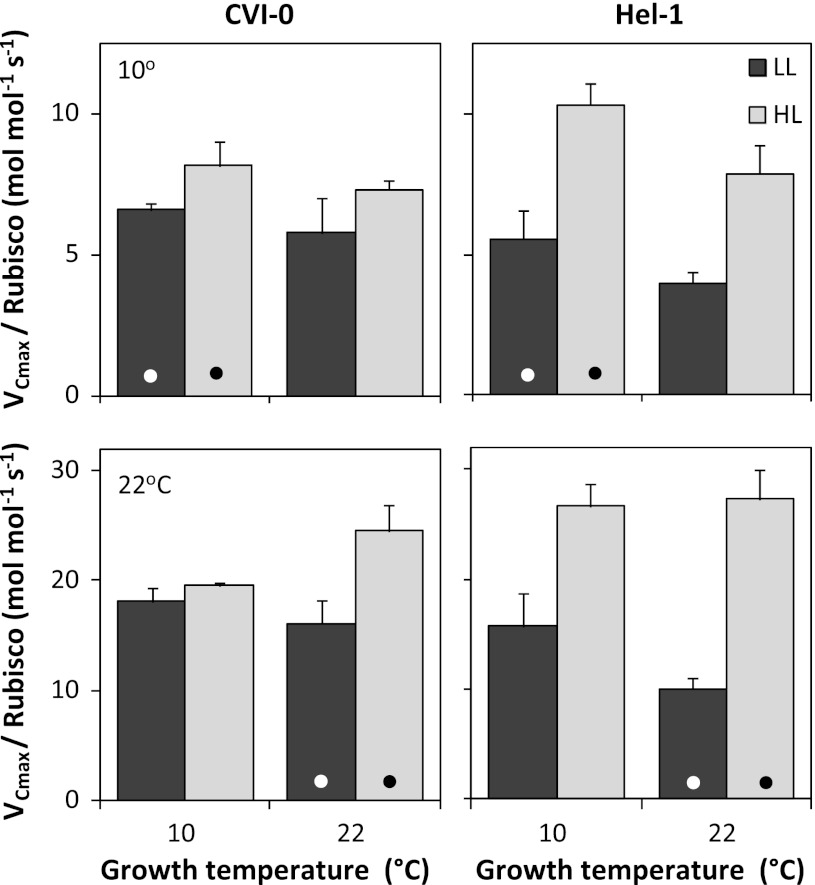
The carboxylation capacity (*V*_Cmax_) expressed per unit Rubisco measured at 10 °C (*upper panels*) and 22 °C (*lower panels*). The *Arabidopsis* accession CVI-0 and Hel-1 were grown at temperatures of 10 °C and 22 °C and irradiances of 50 (LL) and 300 (HL) μmol photons m^−2^ s^−1^. Means + SE are shown (*n* = 3). The *dots* indicate measurements at the growth temperatures

As a consequence, *V*_Cmax_ expressed per unit Rubisco, a measure of the in vivo activity of the carboxylase, was lower at low growth irradiance, particularly in the Hel-1 accession (Fig. 3). Rubisco of LL-plants was probably not fully activated, although photosynthesis was fully induced at the saturating irradiance used for the measurements. Not many reports of this phenomenon are available, but a lower in vivo Rubisco activity was also observed in shaded *Oryza sativa* leaves (Hidema et al. 1991). The reduced *V*_Cmax_ per unit Rubisco contributes to a low efficiency of the utilization of resources for photosynthesis in low irradiance conditions.

*V*_Cmax_ per unit Rubisco was higher in HL-plants when measured at their growth temperatures compared to plants that were not temperature acclimated (Fig. 3). This temperature acclimation effect on in vivo Rubisco activity could be the result of similar changes in in vitro Rubisco specific activity with growth temperature as found for *Spinacia oleracea* (Yamori et al. 2006). Alternatively, the activation state of Rubisco could be reduced in non-acclimated plants, but that was not investigated. As *V*_Cmax_ limits *A*_sat_ at ambient CO_2_ and is determined by Rubisco amount and its specific activity, the maximization of the latter at the growth temperature adds to photosynthetic efficiency. However, this pertains to the high growth irradiance only, as LL-plants did not show a superior *V*_Cmax_ per unit Rubisco at the growth temperature (Fig. 3).

A higher photosynthetic capacity generally requires more mesophyll tissue (Muller et al. 2009; Terashima et al. 2011). A positive relationship between capacity-related variables and leaf mass per unit area (LMA) is thus expected. This was indeed true (Table 2), as the variables pertaining to photosynthetic capacity per unit leaf area, *A*_sat_, *V*_Cmax_ and Rubisco, showed strong correlations with LMA (*r* = 0.95–0.98).

### The balance between light harvesting and photosynthetic capacity

Since chlorophyll content was much less influenced by growth irradiance and temperature (Table 2) compared to *A*_sat_, the latter variable expressed per unit chlorophyll (*A*_sat_/chl) showed a roughly similar response as *A*_sat_ expressed per unit leaf area to growth irradiance and temperature. *A*_sat_/chl measured at a common temperature decreased as a result of higher growth temperature and lower growth irradiance (Table 2). This was most clearly so when measured at 10 °C, whereas the growth temperature effect was small in HL-plants when measured at 22 °C, particularly in the Hel-1 accession (Tables 1, 2). Similar responses were obtained when considering the other capacity-related variables expressed per unit chlorophyll, Rubisco and *V*_Cmax_ (Tables 1, 2). The growth irradiance effects are well known for many species including *Arabidopsis* (Murchie and Horton 1997; Walters et al. 1999; Evans and Poorter 2001; Bailey et al. 2004). The growth temperature effect on capacity variables per unit chlorophyll has not been specifically described for *Arabidopsis*. However, it has been found for cold-tolerant species such as *Plantago asiatica* (Hikosaka 2005), *S.* *oleracea* (Yamori et al. 2005) and *Aucuba japonica* (Muller et al. 2005). Not surprisingly, the cold-tolerant *A.* *thaliana* is also capable of this form of acclimation to temperature.

The shift in the balance between light harvesting and photosynthetic capacity at the chloroplast level, as evident from the capacity-related variables per unit chlorophyll, was also reflected in the chlorophyll *a*/*b* ratio (Tables 1, 2). The low ratio at low growth irradiance and high growth temperature is associated with a large investment in LCHII and thus light harvesting (Anderson et al. 1995; Huner et al. 1998). Photosynthetic rates are necessarily low at a low growth irradiance, which does thus not require much investment in photochemistry. A low growth temperature requires a large investment in the photochemical apparatus to compensate for the reduced enzyme activity. The balance between photon absorption and utilization in photochemistry may be sensed by plants and used for the adjustment to the light and temperature condition (Huner et al. 1998; Bräutigam et al. 2009). The adjustment thus contributes to an efficient utilization of resources for the photosynthetic apparatus.

### The balance between the electron transport and carboxylation capacities

**Fig. 4:**
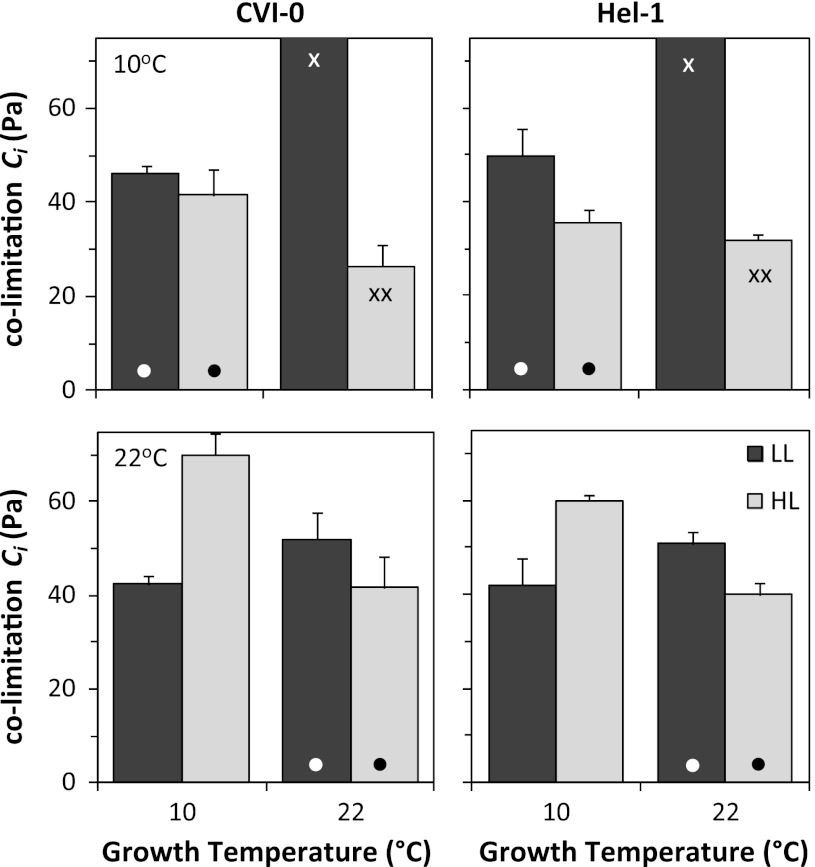
The intercellular CO_2_ partial pressure (*C*_i_) where photosynthesis is co-limited by carboxylation capacity and the regeneration of RuBP (co-limitation *C*_i_) measured at 10 °C (*upper panels*) and 22 °C (*lower panels*). The *Arabidopsis* accession CVI-0 and Hel-1 were grown at temperatures of 10 and 22 °C and irradiances of 50 (LL) and 300 (HL) μmol photons m^−2^ s^−1^. Means + SE (*n* = 3) are shown. The *dots* refer to measurements at the growth temperatures; the *single crosses* indicate that *J*_max_ could not be reliably estimated meaning that the co-limitation *C*_i_ was high; the *double crosses* indicate where photosynthesis at the co-limitation *C*_i_ was not limited by *V*_Cmax_ and *J*_max_ but by *V*_Cmax_ and TPU

The CO_2_ response curves (Fig. 2) were used to derive the carboxylation capacity (*V*_Cmax_) and the electron transport capacity (*J*_max_). The *J*_max_ was difficult to derive from the curves of the HT-plants measured at 10 °C. The HTHL-plants showed a strong limitation by TPU, which prohibited the estimation of *J*_max_, but did not interfere with the estimation of the *C*_i_ where *V*_Cmax_ and RuBP-regeneration co-limit *A*_sat_. Some of the HTLL-plants of both accessions showed no clear transition from the RuBP-saturated to the RuBP-limited range at 10 °C, which indicates that the *J*_max_ must be high relative to *V*_Cmax_, but it prohibited its quantitative estimation. The mean *C*_i_ where *V*_Cmax_ and *J*_max_ co-limit *A*_sat_, further referred to as the co-limitation *C*_i_, was on average 45 Pa at the growth temperatures. This was clearly above the actual *C*_i_ that was on average 29 Pa at the 38 Pa used for the measurements at ambient [CO2] (Figs. 2, 4). The co-limitation *C*_i_ is generally at or slightly above ambient, but some species maintain higher values (Stitt 1991), including *Arabidopsis* as shown here and as also suggested by the data of Tholen et al. (2008). The relatively high co-limitation *C*_i_ indicates that electron transport capacity was larger than necessary at ambient [CO_2_], which decreases resource use efficiency of the photosynthetic apparatus (Hikosaka 1997).

The co-limitation *C*_i_ and the *J*_max_ */V*_Cmax_ ratio were somewhat higher for LL-plants compared to HL-plants for both accessions measured at their growth temperature (Fig. 4; Tables 1, 2). The increase of the *J*_max_ */V*_Cmax_ ratio with decreasing growth irradiance (Table 2) is generally not found in other species (Pons and Pearcy 1994; Poorter and Evans 1998, Hikosaka 2005) but data for *Arabidopsis* are lacking.

The *J*_max_ */V*_Cmax_ ratio decreased at a higher growth temperature in HL-plants (measured at 22 °C), resulting in a similar co-limitation *C*_i_ at the two growth temperatures (Fig. 4; Table 2). The down-regulation of *J*_max_ relative to *V*_Cmax_ at a higher temperature has been described for several species, although not all species show this form of plasticity (Hikosaka et al. 1999; Onoda et al. 2005). *Arabidopsis* growing at high irradiance appears to have this capability of adjustment of the *J*_max_/*V*_Cmax_ ratio to growth temperature also. This adjustment contributes to an increase in resource use efficiency, since *J*_max_ increases stronger with temperature than the initial slope of the CO_2_ response curve (Hikosaka 1997).

Low irradiance grown plants did not show such a down-regulation of *J*_max_ relative to *V*_Cmax_ at a higher growth temperature. On the contrary, the *J*_max_ */V*_Cmax_ and the co-limitation *C*_i_ increased in both accessions, resulting in highly significant interacting effects of temperature and irradiance (Fig. 4; Tables 1, 2). Also the measurement temperature effect was opposite to expected in LL-plants. An increase of the co-limitation *C*_i_ with decreasing measurement temperature was found for these plants (Fig. 4). This increase of the *J*_max_ */V*_Cmax_ ratio in HTLL-plants was even to the extent that *J*_max_ could not be reliably estimated at 10 °C. This measurement and growth temperature effect on the *J*_max_/*V*_Cmax_ ratio in low irradiance grown *Arabidopsis* is difficult to interpret. It cannot be excluded that variation in limitation by the mesophyll conductance for CO_2_ diffusion interfered with the *J*_max_ and *V*_Cmax_ calculations (Ethier and Livingston 2004). Alternatively, the opposite temperature effect on *J*_max_ */V*_Cmax_ at the two growth irradiances could be the result of variation in temperature dependencies of *J*_max_ and/or *V*_Cmax_ with growth irradiance.

### Limitation by triose phosphate utilization

**Fig. 5:**
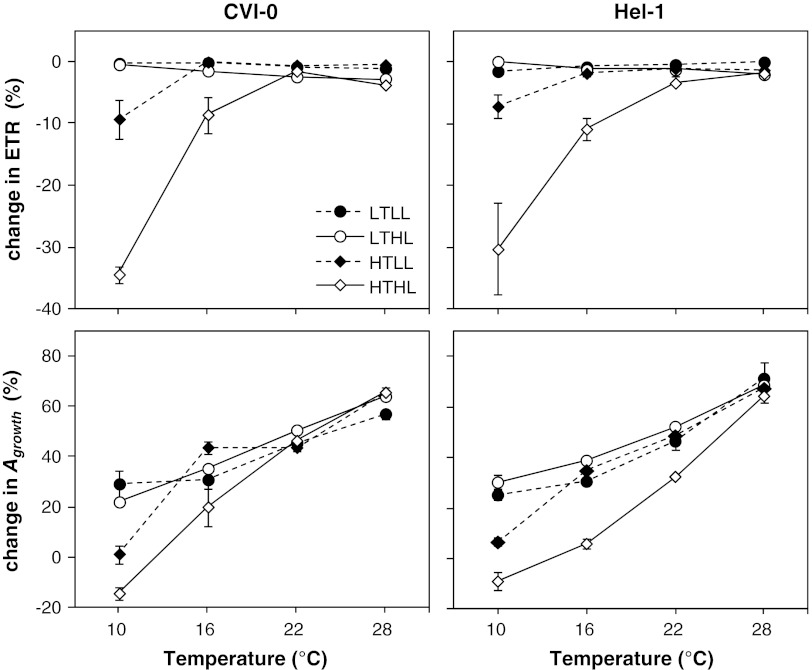
Temperature dependence of the change in photosynthetic rate as a result of a decrease in [O_2_] from 21 % (atmospheric) to 1 % (mean ± SE; *n* = 4). The electron transport rate (ETR; *upper panels*) and the CO_2_ assimilation rate at the growth irradiance (*A*_growth_; *lower panels*) are shown. When limitation by triose-phosphate utilization (TPU) does not play a role, the *A*_growth_ and ETR are expected to increase and to remain constant, respectively. *Symbols* and treatments as in Fig. 1

The O_2_ sensitivity of photosynthesis was used to quantify the temperature dependence of the limitation of photosynthesis by TPU at the growth irradiance. Two measures of the photosynthetic rate were used, *A*_growth_ and ETR. The HT-plants showed no increase of *A*_growth_ upon exposure to 1 % O_2_ at 10 °C and a strong decrease in ETR (Fig. 5). A similar response was evident from the CO_2_ response curves of HTHL-plants that showed no increase of photosynthesis above ambient [CO_2_] (Fig. 2). This clear indication of limitation by TPU diminished when the measurement temperature was increased to 16 °C and was virtually absent at the growth temperature of 22 °C and above. The LT-plants, however, did not show any decrease in ETR across the range of measurement temperatures from 10 to 28 °C in response to a decrease of the O_2_ concentration from 21 to 1 %, nor a less than expected increase of *A*_growth_ (Fig. 5). These plants thus showed no signs of limitation by TPU. Alleviation of TPU limitation with acclimation to cold is well known in *Arabidopsis* (Strand et al. 1997), which is likely to occur by an increase in the capacity of sucrose synthesis (Stitt and Hurry 2002). Growth irradiance effects were generally larger than the effects of growth temperature at the level of the two factor used in the experiments. However, the O_2_ sensitivity of photosynthesis at 10 °C was an exception as the temperature effect was much larger than the irradiance effect for these variables (Tables 1, 2; Fig. 5).

The reduction of ETR and the absence of the increase of *A*_growth_ at low [O_2_] measured at 10 and 16 °C was much less in HTLL-plants compared to HTHL-plants (Fig. 5), which resulted in a highly significant interaction of growth temperature and irradiance at 10 °C (Table 1). Remarkably, the CO_2_ response curves of HTLL-plants measured at 10 °C showed no indication of limitation by TPU (Fig. 2). This inconsistency between the two measures of TPU limitation is difficult to explain. Nevertheless, it is clear that the limitation by TPU at temperatures lower than 22 °C was less in low compared to high irradiance grown HT-plants. Apparently, the HTHL *Arabidopsis* operated at a capacity of triose-phosphate processing that is close to the supply from the chloroplast in the growth conditions, whereas HTLL-plants had a larger capacity relative to the supply. This growth irradiance effect is unknown. The larger capacity of triose-phosphate processing relative to its supply requires investments that is not utilized in the growth conditions, and thus further contributes to inefficient utilization of available resources for leaf functioning at low irradiance in *Arabidopsis*.

### Comparison of the two accessions

Growth temperature and irradiance effects were much stronger than the differences between the two accessions, if there were any. This is evident from the high* F* values for particularly the irradiance effects. *F* values for the accession effects were low and not significant in many cases (Table 1). Significant differences that were found include the following (Table 2). Chlorophyll contents and LMA in high temperature grown CVI-0 were higher than for Hel-1. The temperature and irradiance effects on *V*_Cmax_ were somewhat stronger in Hel-1. The growth temperature effect on *A*_sat_ per unit chlorophyll was somewhat stronger in CVI-0 and the irradiance effect on *V*_Cmax_ per chlorophyll was somewhat stronger in Hel-1. These two capacity variables per chlorophyll were measured on different sets of leaves, which is likely to be the reason for these slightly different temperature and irradiance effects. The conclusion is that the two accessions were remarkably similar in their acclimation to the combination of temperature and irradiance.

Differences were expected in the comparison of CVI-0 and Hel-1 that originate from such widely different climates. The small differences that were found are not consistent with the expectation that the CVI-0 accession has a better capability of photosynthetic acclimation to high irradiance, and the Hel-1 accession to low temperature and/or low irradiance. The number of accessions is not sufficient to draw definitive conclusions on the absence of climatic differentiation in photosynthetic adaptation in *Arabidopsis*. However, if these two accession are representative, then its absence would contrast with, e.g., *Solidago virgaurea* that showed differences between ecotypes in acclimation to irradiance (Björkman and Holmgren 1963), *Atriplex lentiformis* with ecotypic differentiation in temperature acclimation (Pearcy 1977), and *Plantago* *asiatica* that showed some intraspecific altitudinal variability in plasticity of the *J*_max_ */V*_Cmax_ ratio (Ishikawa et al. 2007). It would also contrast with other traits of *Arabidopsis* as among others pertaining to seed dormancy and flowering time (Koornneef et al. 2004; Stinchcombe et al. 2004), differentiation at the molecular level (Hancock et al. 2011), and chromatin compaction (Tessadori et al. 2009) that appeared to be associated with climate. These results suggest that differentiation in adaptation of the photosynthetic apparatus to climate is not well developed in *Arabidopsis*. This tentative conclusion awaits confirmation from a broader comparison including a larger number of ecotypes.

## Conclusions

*Arabidopsis* showed photosynthetic acclimation to temperature and irradiance as is in line with what has been reported previously for this and various other species. However, several variables used to evaluate the acclimation showed interacting effects of the two environmental factors. The relative effect of growth temperature on photosynthetic capacity variables (*A*_sat_/LA, *A*_sat_/chl, *V*_Cmax_/LA, *V*_Cmax_/chl) was smaller in plants grown at high compared to low irradiance. Hence, acclimation to temperature of these aspects of photosynthetic functioning depends on growth irradiance. However, evaluation of the interaction depends on measurement temperature, since it was only evident at 22 °C and not at 10 °C. This contrasted with the stronger temperature effect on photosynthetic rate (*A*_growth_ and ETR) of high irradiance grown plants measured at 10 °C (but not at 22 °C), which could be explained from the different role of light limitation in the different temperature and irradiance conditions.

HT-plants showed the normally found decrease of the *J*_max_ */V*_Cmax_ ratio with increasing temperature. However, LT-plants displayed unexplained growth and measurement temperature effects on *J*_max_ */V*_Cmax_ and thus the *C*_i_ where co-limitation occurs between photosynthesis limited by Rubisco and by regeneration of RuBP. *V*_Cmax_ that limited *A*_sat_ at ambient [CO_2_] was low in LL-plants when expressed per unit Rubisco. The low irradiance grown plants compared to the ones grown at high irradiance showed also a lesser limitation by TPU. These traits contribute to a low efficiency of the use of resources for photosynthesis of *Arabidopsis* growing in low irradiance conditions.

Differences in the capability of photosynthetic acclimation to temperature and irradiance were expected for the two *Arabidopsis* accessions from contrasting climates. However, they showed remarkably similar temperature and irradiance effects on the variables included in this study. Climatic differentiation in photosynthetic variables that can be interpreted as adaptation of the photosynthetic apparatus in Arabidopsis was thus not evident in the present comparison.
